# Interspecific Nuclear Transfer Blastocysts Reconstructed from Arabian Oryx Somatic Cells and Domestic Cow Ooplasm

**DOI:** 10.3390/vetsci10010017

**Published:** 2022-12-28

**Authors:** Aiman A. Ammari, Muath G. ALGhadi, Ramzi A. Amran, Nawal M. Al Malahi, Ahmad R. Alhimaidi

**Affiliations:** Department of Zoology, College of Science, King Saud University, P.O. Box 2455, Riyadh 11451, Saudi Arabia

**Keywords:** iSCNT, cloning, Arabian Oryx, blastocyst

## Abstract

**Simple Summary:**

Interspecies SCNT-based cloning and in vitro production of interspecies SCNT-derived embryos allows cells that have undergone terminal differentiation to be reprogrammed to become totipotent cells. The development of the interspecific Somatic cell nuclear transfer oryx in vitro for the first time in Saudi Arabia will promote additional research on animal reproductive cloning for the preservation of endangered species.

**Abstract:**

Cloning, commonly referred to as somatic cell nuclear transfer (SCNT), is the technique of enucleating an oocyte and injecting a somatic cell into it. This study was carried out with interspecific SCNT technology to clone the Arabian Oryx utilizing the oryx’s fibroblast cells and transfer it to the enucleated oocytes of a domestic cow. The recipient oocytes were extracted from the cows that had been butchered. Oryx somatic nuclei were introduced into cow oocytes to produce embryonic cells. The study was conducted on three groups, Oryx interspecific somatic cell nuclear transfer into enucleated oocytes of domestic cows, cow SCNT “the same bovine family species”, used as a control group, and in vitro fertilized (IVF) cows to verify all media used in this work. The rates of different embryo developmental stages varied slightly (from 1- cell to morula stage). Additionally, the oryx interspecies Somatic cell nuclear transfer blastocyst developmental rate (9.23%) was comparable to that of cow SCNT (8.33%). While the blastula stage rate of the (IVF) cow embryos exhibited a higher cleavage rate (42%) in the embryo development stage. The results of this study enhanced domestic cow oocytes’ ability to support interspecific SCNT cloned oryx, and generate a viable embryo that can advance to the blastula stage.

## 1. Introduction

By the early 1970s, the Arabian Oryx had become extinct in the wild but was still thriving in zoos and private preserves. The Arabian Oryx was the first animal to be designated as vulnerable in 2011 after being listed as extinct in the wild and subsequently as endangered in 1986 on the International Union for Conservation of Nature’s (IUCN) Red List. The Arabian oryxes are classified as belonging to the family Bovidae, subfamily Hippotragini, and genus Antelope [1,2,3]. In Saudi Arabia, the National Wildlife Research Center (NWRC) under the Saudi Wildlife Commission (SWC) launched an oryx conservation and restoration effort in 1989. Simultaneous conservation initiatives for the preservation of large expanses within the original Arabian Oryx range, as well as the breeding of oryx in captivity at the NWRC in Taif [4]. The karyotype of these species has already been identified. The diploid chromosome number of the Arabian oryx, Oryx leucoryx, is 2n = 58 [5,6]. Since somatic cell nuclear transfer was utilized to successfully clone Dolly the sheep in 1997, other researchers have used these methods to clone both domestic and wild animals. Through the development of the so-called interspecies somatic cell nuclear transfer technique over the past decade, it is now possible to clone endangered species of animals. Many researchers have conducted successful experiments on cloning mammals of the same species have published their findings, including sheep [7,8]; rabbits [9,10]; pigs [11]; mice [12]; monkeys [13]; goats [14,15]; and camels [16]. These studies opened new horizons for scientists to continue cloning research, such as interspecies and interspecific cloning of Bos gaurus into bovines [17]; various mammal species into bovine [18]; Bos gaurus into bovine [19,20]; Bos javanicus into bovine [21]; bovine-ovine [22]; cat into bovine [23,24]; cattle, goat, and cat into Bubalus bubalis buffalo [25]; cattle and pig [26]; cat into cow [27]; cat into cow [28]; human into bovine [29]; human into rabbit [30], Asiatic cheetah into cat [31]; human into bovine [32]; human into rabbit [33]; argali (Ovis ammon into sheep) [34]. The ability of donor cell nuclei to undergo epigenetic changes in the cytoplasm of rebuilt oocytes [35,36,37] and the prevalence of programmed cell death in nuclear donor cells and cloned embryos are two major factors that appear to have a significant impact on SCNT efficiency [38,39,40]. Epigenetic reprogramming is regarded to be the main factor behind most developmental abnormalities in clones [41]. Challenges including mitochondrial/genomic DNA compatibility, activation of the donor cell’s embryonic genome by the recipient oocyte, and a lack of suitable foster mothers for interspecific somatic cell nuclear transfer embryos prevent the generation of interspecific somatic cell nuclear transfer animals [42]. Despite substantial challenges, SCNT is frequently viewed as a way to save endangered species or bring back extinct species. Every few years, whenever some progress has been achieved in the topic, the “de-extinction” of the mammoth is discussed in popular media [43]. The Pyrean Ibex was successfully cloned using tissue from the last known available specimen; however, the newborn passed away from left lung atelectasis soon after delivery. most of the thoracic cavity [44]. Over many decades, it has been acknowledged that the biggest obstacle to a cloned embryo’s development is its epigenetic reprogramming, which can cause problems with implantation, placenta development, and function, as well as obesity, immunodeficiency, respiratory defects, and early death [45,46]. It has been determined that the low birth rate is largely due to this epigenetics problem [47].

Cloned animals do not have identical somatic mtDNA copies while sharing the same nuclear DNA. As a result, they are not exact clones of their somatic cell nucleus ancestors. Moreover, recipient oocytes are frequently extracted from the ovaries of females who have been slaughtered, enabling a high-quality positive selection. However, these oocytes have unknown genetic backgrounds and unknown cytoplasmic maternal lineage. Due to the variability of certain mtDNA nucleotide sequences, clonal embryos and their progeny frequently exhibit a high level of mitochondrial genotype heterogeneity if the recipient nuclei are not derived from the same maternal lineage as the donor nuclei. Using cadaver somatic cells, the endangered grey wolf has been successfully cloned [48]. In wild goats, somatic cells were inserted into the oocyte of a local goat [49]. Somatic cell cloning efficiency is typically extremely low, even though somatic cell nuclear transfer has produced cloned offspring in a number of mammalian species through intra- or inter-species somatic cell nuclear transfer. Therefore, further investigation is required to identify the factors governing SCNT-mediated cloning that might be responsible for enhancing the efficacy of this contemporary assisted reproductive method. The aim of this study is to clone the Arabian oryx utilizing in vitro interspecific somatic cell nuclear transfer, which entails inserting an oryx somatic cell into a domestic cow enucleated egg and comparing it to the in vitro development of the cow SCNT embryo.

## 2. Materials and Methods

### 2.1. Chemicals

Unless otherwise stated, all chemicals, including hormones, came from Sigma Aldrich Corp., St. Louis, MO, USA.

### 2.2. Ovaries, Oocyte, and Maturation

From 1 January to 31 August 2022, all cow ovaries were provided by the Riyadh local slaughterhouse and transported in 0.9% NaCl over a period of 1 to 2 h to the laboratory unit of Embryonic studies and reproductive physiology at King Saud University. The removal of oocytes from the ovarian follicles on the ovary’s roof was carried out using a disposable syringe with a 10 mL vial and 0.5 mL of handling medium (TCM 199 (Hanks solution, fetal bovine serum (10%), Gentamycin, Na—Pyruvate from Stock Solution (100 mM) (Caisson Lab. Inc., Smithfield, UT, USA)). After maturing in the lab for 24 h, cumulus–oocyte complex (1832) showed uniform cytoplasm and more than three layers of cumulus cells [50].

### 2.3. Dissect Zona with Micropipettes

After maturation, cumulus-enclosed oocytes were placed in a petri dish with a solution of the enzyme hyaluronidase (600 IU/2 mL). The cumulus cells were manually extracted with a glass Pasteur pipette. Only eggs with an extruded first polar body (FPB) were assessed for zona dissection. By placing the oocyte on the holding pipette with the polar body orientation at 12 o’clock, the cutting pipette was used to cut a slot or slit in the ZP on a different region of the oocyte [7,51].

### 2.4. Oocyte Enucleation

**Figure 1:**
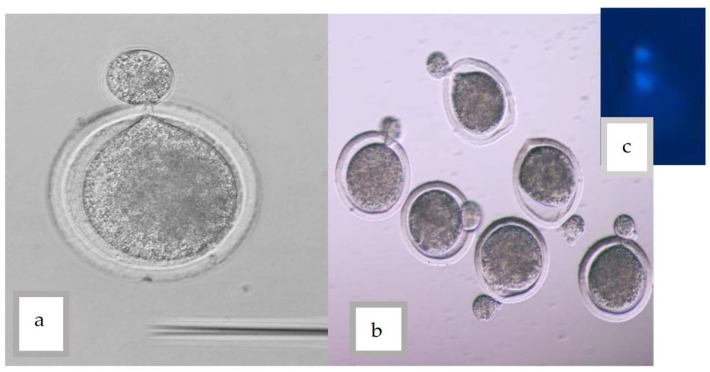
(**a**,**b**) A small amount of cytoplasm was pushed out from the ZP (**c**) Confirmation of enucleation using Hoechst stain and UV light.

By gently rotating the oocyte with cutting pipettes, a small amount of cytoplasm that is situated in the plasma membrane is excluded just below the first polar body of oocytes outside the zona pellucida (Figure 1). By removing the resulting cytoplasm from the oocyte and preferentially culturing it in TCM-199, to determine the enucleation of the oocytes. For 30 min, Earle’s salt, 10% FBS, and 10 μg/1 mL Hoechst are combined (Sigma B2261). Subsequently, ultraviolet (UV) light was used to examine the ooplasm to see if it had reached the metaphase II stage.

### 2.5. Preparation of Fibroblast from Arabian Oryx

Through our previous study [51], the appropriate passage number was identified, which is used in our current study.

### 2.6. Nuclear Transfer, Fusion, and Activation

A fibroblast cell from an oryx was injected into the perivitelline space employing polar body dissection pipettes. To achieve cell fusion immediately following nuclear transfer (NT) at room temperature, a single alternating current pulse of 0.2 Kv/c for one second was applied, followed by a single DC pulse of 2.5 Kv/c for fifty seconds. Moreover, the oocyte was artificially activated by being exposed to 5 M ionomycin right away after it had been fused. The incubation was carried out in synthetic oviductal fluid (SOF) medium supplemented with 1 mg/250 BSA for 6 h at 39 °C and 5% CO_2_, 10 μL/mL MEM (50×), 10 μL/mL MEM (100×), g/mL penicillin-streptomycin, and 2.5 μg/250 μL cycloheximide on a 35 mm Petri dish with mineral oil.

### 2.7. In Vitro Culture of iSCNT Embryos

Interspecific SCNT embryos were activated for 6 h before being cultured for 42 h in SOF medium with 1 mg/250 μL BSA, 10 μL/1 mL MEM (50×), and 10 μL/mL MEM (100×), μg/2 mL penicillin-streptomycin, in 35 mm Petri dishes with mineral oil. Subsequently, the embryos were cultured for 6 h at 39 °C with a mixed gas of 90% N_2_, 5% CO_2_, and 5% O_2_ [7,51].

### 2.8. Statistical Analysis

The Mine Tab INSTAT software was utilized to record and examine all data. The Chi-square analysis and sample *t*-test were employed to examine the cleavage and blastocyst rates.

## 3. Results

**Figure 2:**
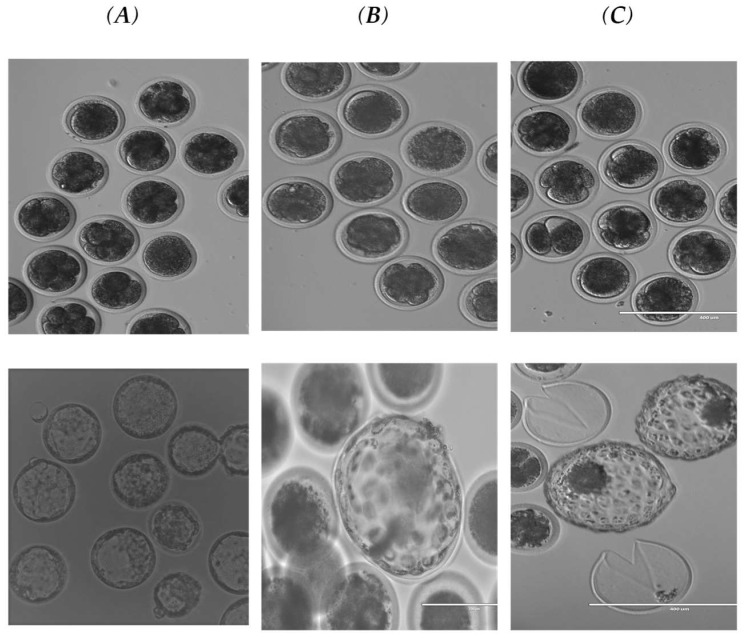
Examples of Morula and blastocyst produced in each group. (**A**): (IVF), (**B**): (interspecific somatic cell nuclear transfer), and (**C**): (SCNT).

A total of 1832 oocytes were collected from slurred cow ovaries, including 810 oocytes from interspecific somatic cell nuclear transfer, 716 oocytes from the cow (SCNT), and 306 from cow IVF. The result of the Oryx reconstructed oocytes compared to cow SCNT, and cow IVF is shown in Table 1. The results showed that the total no of matured oocytes with the first polar body (1st PB) oocytes used for both Oryx and cow (SCNT) cloning (1064/1526 = 69.72%) oocytes. Total enucleated oocytes, (890/1064 = 77.1%), with a total number of activated oocytes for both Oryx and cow (SCNT) (540/890 = 60.67% oocytes). A total of 839 embryos were cultured, including 241 embryos for Oryx, 292 embryos for SCNT, and 306 embryos for IVF. The total cleavage embryo development from the 2-cellsstage up to the blastula stage (Figure 2) of both Oryx (26.97%) and cow (SCNT) (33.0%) with no significant differences between them (Table 1). There were some variations between the different stages of embryo development (from 1-cell up to the morula stage). The 1-cell stage of Oryx (20.52%) and (26.14%) for cow IVF showed a higher arrest stage than cow (SCNT) (4.41%), Table 2. The 2-cell stage embryos of the Oryx cloned embryo interspecific somatic cell nuclear transfer and cow (SCNT) showed a similar rate and their total rate (29.75%), which showed a higher developmental rate than the IVF rate (12.15%). While the 4-cells stage of the Oryx showed a higher developmental rate (29.23%) than the cow (SCNT) (6.54%) and cow IVF (7.48%). In comparison, the developmental rate of the 8-cells stage of both cloned Oryx and cow (SCNT) embryos’ developmental stage (21.52%) was higher than that of the cow IVF (7.48%). The 16-cell stage cloned cow embryo showed a higher rate (19.35%) compared to the Oryx (3.08%) and cow IVF (0%) rates. The rates at the morula stage of the cloned embryos (15.06%) and IVF (11.21%) are close to each other and are higher than the Oryx rate (6.15%), Table 2. Finally, the blastocyst developmental rate of Oryx interspecific rate (9.23%) group 1 showed a close blastocyst development rate to that of the cow (SCNT) group 2 (8.33%). While the 3rd group, the (IVF) cow embryos, showed a cleavage rate (42%) of the embryo development stage, with a blastula stage rate (57%), which is a significantly higher (*p* < 0.05) higher than the cloned embryo rate (Table 1 and Table 2).

## 4. Discussion

Although there is a growing desire to use cloning to preserve endangered species, effective interspecies nuclear transfer has yet to be reported as SCNT within the same species. Based on the outcomes of this research, cow oocytes enable the development of Arabian Oryx embryos’ interspecific somatic cell nuclear transfer up until the blastocyst stage, as stated in our previous paper [51]. Early embryogenesis is a critical stage of embryo development, and its success depends on the ability of the nucleus and the cytoplasm to communicate effectively. Since the donor nucleus and the recipient cytoplasm are from different species in interspecific somatic cell nuclear transfer, the challenge is significantly more complicated. The majority of the interspecific somatic cell nuclear transfer embryos were found to stop developing at the recipient cytoplast-specific embryonic genome activation (EGA) stage [52]. As per the findings of this study, Cow–Oryx interspecific somatic cell nuclear transfer embryos can progress past the EGA stage, and about 9% of them survived until the blastocyst stage, which is comparable to the rate in most SCNT studies. This showed that the cow and Oryx have a healthy nucleus–cytoplasm connection. The results of our study showed superior results to those obtained in other studies of interspecific somatic cell nuclear transfer in different animals, such as [25,28,52,53,54,55,56,57]. Producing animals by interspecific somatic cell nuclear transfer presents a variety of challenges, including incompatibility of mitochondrial/genomic DNA, activation of the nucleus’ embryonic genome by the donor oocyte, the lack of fosters who are appropriate for interspecific somatic cell nuclear transfer embryos, and epigenetic disorders and unstable gene expression formations [58,59]. When compared to interspecies cloning, intraspecies cloning is more likely to have higher development efficiency the closer the donor cell’s species is to the recipient oocyte [19]. Cow oocytes have been employed in numerous investigations as extremely effective recipient cytoplasts for their capacity to reprogram somatic cells. This is consistent with both our findings and with other species [17,18,20,21,29,60,61]. Since Multiple Barrier Removal techniques have the potential to eliminate the demand for any conventional pharmaceutical therapy but may require extra injections, they may hold the key to the future of SCNT. Once again, blastocyst development or cleavage rate, which are usually mentioned as success indicators in research, may not be significantly correlated with the survivability of the embryo during pregnancy. Even though there is a lot of promising research, their true value can only be gauged by how many healthy offspring they can produce [62]. One of the factors that has been connected to cloning failure is a higher rate of oocyte degradation. Given that the majority of the donor cells came from skin cumulus cells, which are not at the G0 stage, this could be because of the type of somatic cell employed, or it could be because of the electrical current used to fuse the somatic cell with the oocytes, which could damage the ova’s cytoplasm. Furthermore, due to the fibroblast cell’s prolonged lifespan, in vitro culture may affect the rate of SCNT formation [63]. These findings imply that techniques for restoring extinct or critically endangered species and populations may involve somatic cell cloning. Even though cloning is not a practical conservation approach in Saudi Arabia, we are nevertheless hopeful that it will aid in preserving endangered species in the future.

## 5. Conclusions

The results of our study indicated that the development of Arabian Oryx interspecific somatic cell nuclear transfer embryos was supported by cow oocytes up to the blastocyst stage. This showed that the cow and Oryx have a healthy nucleus–cytoplasm connection. This gave us a good impression of the ability of cow oocytes as recipient cytoplasts, highly efficient in their ability to reprogram Oryx somatic cells. Therefore, additional studies are necessary to ascertain how this affects the interspecific somatic cell nuclear transfer success rate. Here, a logical conclusion must be derived: for cloning to be more successful, it will be necessary to have a greater understanding of oocyte physiology—not just as it pertains to mammalian oocytes, but also as it relates to species-specific specialization and uniqueness. More study is required, especially on the intricate topic of how different types of injectable medications interact with different nucleus–donor cell types. These findings imply that somatic cell cloning techniques can restore extinct or critically endangered species and populations. Even though cloning is not a practical conservation approach in Saudi Arabia, we are nevertheless hopeful that it will one-day aid endangered species.

## Figures and Tables

**Table 1:** The number of cow oocytes used for cloning Oryx and cow-cleaved embryos of interspecific somatic cell nuclear transfer, (SCNT) and in vitro fertilization (IVF).

Group	No. of Oocytes No, %	With FB No, %	Without FB No, %	No. of Enucleation No, %	No. of Activation No, %	No. of Culture Embryo%	Cleavage Rate % No, %	Blastocyst Rate % No, %
Oryx interspecific SCNT	810/1832 = 44.21%	536/810 = 66.17%	274/810 = 33.83%	439/536 = 81.90%	241/439 54.90%	150/241 = 62.24%	65/241 = 26.97%	6/65 = 9.23%
Cow SCNT	716/1832 = 39.09%	528/716 = 73.74%	188/716 = 26.26%	451/528 = 85.41%	299/451 = 66.30%	230/299 = 76%	96 (33%)	8/96 = 8.33%
Cow IVF	306/1832 = 16.7%	-	-	-	-	306	130 (42%)	74 (57%)

Percentages of cleavage and blastocysts obtained.

**Table 2:** The comparison of the cloned Oryx interspecific somatic cell nuclear transfer, Cow SCNT, and IVF of cow embryo developmental stages.

Type	Oocytes No.	With FB No. & %	1 Cell No. & %	2 Cells No. & %	4 Cells No. & %	8 Cells No. & %	16 Cells No. & %
Oryx interspecific SCNT	810	536/810 = 66.17%	110/536 = 20.52%	21/65 = 32.31%	19/65 = 29.23%	13/65 = 20%	2/65 = 3.08%
Cow SCNT	716	522/716 = 72.91%	23/522 = 4.41%	26/93 = 27.96%	6/93 = 6.45%	21/93 = 22.58%	18/93 = 19.35%
IVF cow	306	-	80/306 = 26.14%	13/107 12.15%	8/107 = 7.48%	8/107 = 7.48%	-

## Data Availability

Not applicable.

## References

[1] Nowak R.M., Paradiso J.L. (1983). Mustela erminea. Walker’s Mammals of the World.

[2] Spinage C.A. (1986). Natural History of Antelopes.

[3] Wilson D.E., Reeder D.M. (2005). Mammal Species of the World: A Taxonomic and Geographic Reference.

[4] Islam M.Z., Ismail K., Boug A. (2011). Restoration of the endangered Arabian Oryx Oryx leucoryx, Pallas 1766 in Saudi Arabia lessons learnt from the twenty years of re-introduction in arid fenced and unfenced protected areas: (Mammalia: Artiodactyla). Zool. Middle East.

[5] Wurster D.H., Benirschke K. (1968). Chromosome studies in the superfam~ly Bovoidea. Chromosoma.

[6] Newnham R.E., Davidson W.M. (1967). The karyotype of the South Arabian oryx, ONX ICUCOI~(PXa llas), Mamma l Chmm Ncw.sI. Oryx Leucoryx.

[7] Al-Ghadi M.Q., Alhimaidi A.R., Iwamoto D., Al Mutary M.G., Ammari A.A., Saeki K.O. (2020). The in vitro development of cloned sheep embryos treated with Scriptaid and Trichostatin (A). Saudi J. Biol. Sci..

[8] Bondioli K.R., Westhusin M.E., Looney C.R. (1990). Production of identical bovine offspring by nuclear transfer. Theriogenology.

[9] Collas P., Balise J.J., Robl J.M. (1992). Influence of cell cycle stage of the donor nucleus on development of nuclear transplant rabbit embryos. Biol. Reprod..

[10] Qu P., Shen C., Du Y., Qin H., Luo S., Fu S. (2020). Melatonin protects rabbit somatic cell nuclear transfer (SCNT) embryos from electrofusion damage. Sci. Rep..

[11] Prather R.S., Sims M.M. (1989). First NL. Nuclear transplantation in early pig embryos. Biol. Reprod..

[12] Kamimura S., Inoue K., Mizutani E., Kim J.M., Inoue H., Ogonuki N. (2021). Improved development of mouse somatic cell nuclear transfer embryos by chlamydocin analogues, class I and IIa histone deacetylase inhibitors. Biol. Reprod..

[13] Meng L., Ely J.J., Stouffer R.L., Wolf D.P. (1997). Rhesus monkeys produced by nuclear transfer. Biol. Reprod..

[14] Hajian M., Jafarpour F., Aghamiri S.M., Varnosfaderani S.R., Esfahani M.H.N. (2020). Effects of ovary storage temperature and embryo vitrification on somatic cell nuclear transfer outcomes in goats. Reprod. Fertil..

[15] Skrzyszowska M., Samiec M. (2021). Generating cloned goats by somatic cell nuclear transfer—Molecular determinants and application to transgenics and biomedicine. Int. J. Mol. Sci..

[16] Wani N.A., Wernery U., Hassan F.A.H., Wernery R., Skidmore J.A. (2021). Production of the first cloned camel by somatic cell nuclear transfer. Biol. Reprod..

[17] Lanza R.P., Cibelli J.B., Diaz F., Moraes C.T., Farin P.W., Farin C.E., Hammer C.J., West M.D., Damiani P., Damiani P. (2000). Cloning of an endangered species (*Bos gaurus*) using interspecies nuclear transfer. Cloning.

[18] Dominko T., Mitalipova M., Haley B., Beyhan Z., Memili E., McKusick B., First N.L. (1999). Bovine oocyte cytoplasm supports the development of embryos produced by nuclear transfer of somatic cell nuclei from various mammalian species. Biol. Reprod..

[19] Li Y., Dai Y., Du W., Zhao C., Wang H., Wang L., Li R., Liu Y., Wan R., Li N. (2006). Cloned endangered species takin (*Budorcas taxicolor*) by inter-species nuclear transfer and comparison of the blastocyst development with yak (*Bos grunniens*) and bovine. Mol. Reprod. Dev. Inc. Gamete Res..

[20] Li Y., Li S., Dai Y., Du W., Zhao C., Wang L., Wang H., Li R., Liu Y., Wan R. (2007). Nuclear reprogramming in embryos generated by the transfer of yak (*Bos grunniens*) nuclei into bovine oocytes and comparison with bovine–bovine SCNT and bovine IVF embryos. Theriogenology.

[21] Sansinena M.J., Hylan D., Hebert K., Denniston R.S., Godke R.A. (2005). Banteng (*Bos javanicus*) embryos and pregnancies produced by interspecies nuclear transfer. Theriogenology.

[22] Hua S., Zhang Y., Song K., Song J., Zhang Z., Zhang L., Zhang C., Cao J., Ma L. (2008). Development of bovine–ovine interspecies cloned embryos and mitochondria segregation in blastomeres during preimplantation. Anim. Reprod. Sci..

[23] Thongphakdee A., Kobayashi S., Imai K., Inaba Y., Tasai M., Tagami T., Nirasawa K., Nagai T., Saito N., Techakumphu M. (2008). Interspecies nuclear transfer embryos reconstructed from cat somatic cells and bovine ooplasm. J. Reprod. Dev..

[24] Thongphakdee A., Numchaisrika P., Omsongkram S., Chatdarong K., Kamolnorranath S., Dumnui S., Techakumphu M. (2006). In vitro development of marbled cat embryos derived from interspecies somatic cell nuclear transfer. Reprod. Domest. Anim..

[25] Selokar N.L., George A., Saha A.P., Sharma R., Muzaffer M., Shah R.A., Palta P., Chauhan M.S., Manik R.S., Singla S.K. (2011). Production of interspecies handmade cloned embryos by nuclear transfer of cattle, goat, and rat fibroblasts to buffalo (*Bubalus bubalis*) oocytes. Anim. Reprod. Sci..

[26] Takeda K. (2013). Mitochondrial DNA transmission and confounding mitochondrial influences in cloned cattle and pigs. Reprod. Med. Biol..

[27] Wittayarat M., Sato Y., Do L.T.K., Chatdarong K., Tharasanit T., Techakumphu M., Taniguchi M., Otoi T. (2017). Epigenetic modulation on cat-cow interspecies somatic cell nuclear transfer embryos by treatment with trichostatin A. Anim. Sci. J..

[28] Do L.T.K., Wittayarat M., Sato Y., Chatdarong K., Tharasanit T., Techakumphu M., Hirata M., Tanihara F., Taniguchi M., Otoi T. (2021). Comparison of Blastocyst Development between Cat-Cow and Cat-Pig Interspecies Somatic Cell Nuclear Transfer Embryos Treated with Trichostatin A. Biol. Bull..

[29] Chang K.H., Lim J.M., Kang S.K., Lee B.C., Moon S.Y., Hwang W.S. (2003). Blastocyst formation, karyotype, and mitochondrial DNA of interspecies embryos derived from the nuclear transfer of human cord fibroblasts into enucleated bovine oocytes. Fertil. Steril..

[30] Chen Y., He Z.X., Liu A., Wang K., Mao W.W., Chu J.X., Lu Y., Fang Z.F., Shi Y.T., Yang Q.Z. (2003). Embryonic stem cells generated by nuclear transfer of human somatic nuclei into rabbit oocytes. Cell Res..

[31] Moulavi F., Hosseini S.M., Tanhaie-Vash N., Ostadhosseini S., Hosseini S.H., Hajinasrollah M., Asgharia M.H., Gourabi H., Shahverdi A., Vosough A.D. (2017). Interspecies somatic cell nuclear transfer in Asiatic cheetah using nuclei derived from post-mortem frozen tissue in absence of cryo-protectant and in vitro matured domestic cat oocytes. Theriogenology.

[32] Carvalho B.P., Cunha A.T., Silva B.D., Sousa R.V., Leme L.O., Dode M.A., Melo E.O. (2019). Production of transgenic cattle by somatic cell nuclear transfer (SCNT) with the human granulocyte colony-stimulation factor (hG-CSF). J. Anim. Sci. Biotechnol..

[33] Ji J., Guo T., Tong X., Luo L., Zhou G., Fu Y., Liu Y. (2007). Experimental cloning of embryos through human-rabbit inter-species nuclear transfer. Front. Biol..

[34] Pan X., Zhang Y., Guo Z., Wang F. (2014). Development of interspecies nuclear transfer embryos reconstructed with argali (*Ovis ammon*) somatic cells and sheep ooplasm. Cell Biol. Int..

[35] Samiec M., Skrzyszowska M. (2018). Can reprogrammingof overall epigenetic memory and specific parental genomicimprinting memory within donor cell-inherited nuclear ge-nome be a major hindrance for the somatic cell cloning ofmammals? A review. Ann. Anim. Sci..

[36] Wiater J., Samiec M., Skrzyszowska M., Lipiński D. (2021). Trichostatin A-assisted epigenomic modulation affects the expression profiles of not only recombinant human α1, 2-fucosyltransferase and α-galactosidase A enzymes but also Galα1→ 3Gal epitopes in porcine bi-transgenic adult cutaneous fibroblast cells. Int. J. Mol. Sci..

[37] Zhou C., Zhang J., Zhang M., Wang D., Ma Y., Wang Y., Wang Y., Huang Y., Zhang Y. (2000). Transcriptional memory inherited from donor cells is a developmental defect of bovine cloned embryos. FASEB J..

[38] Samiec M., Romanek J., Lipinski D., Opiela J. (2019). Expression of pluripotency-related genes is highly dependenton trichostatin A-assisted epigenomic modulation of porcinemesenchymal stem cells analysed for apoptosis and subse-quently used for generating cloned embryos. Anim. Sci. J..

[39] Wang M., Gao Y., Qu P., Qing S., Qiao F., Zhang Y., Mager J., Wang Y. (2017). Sperm-borne miR-449b in-fluences cleavage, epigenetic reprogramming and apoptosisof SCNT embryos in bovine. Sci. Rep..

[40] Zhang Y., Qu P., Ma X., Qiao F., Ma Y., Qing S., Zhang Y., Wang Y., Cui W. (2018). Tauroursodeoxycholicacid (TUDCA) alleviates endoplasmic reticulum stress of nuclear donor cells under serum starvation. PLoS ONE.

[41] Gouveia C., Huyser C., Egli D., Pepper M.S. (2020). Lessons learned from somatic cell nuclear transfer. Int. J. Mol. Sci..

[42] Loi P., Modlinski J.A., Ptak G. (2011). Interspecies somatic cell nuclear transfer: A salvage tool seeking first aid. Theriogenology.

[43] Loi P., Saragusty J., Ptak G. (2014). Cloning the mammoth: A complicated task or just a dream?. Adv. Exp. Med. Biol..

[44] Folch J., Cocero M.J., Chesne P., Alabart J.L., Domínguez V., Cognie Y., Roche A., Fernández-Árias A., Martí J., Sánchez P. (2009). First birth of an animal from an extinct subspecies (*Capra pyrenaica pyrenaica*) by cloning. Theriogenology.

[45] Niemann H. (2016). Epigenetic reprogramming in mammalian species after SCNT-based cloning. Theriogenology.

[46] Loi P., Iuso D., Czernik M., Ogura A. (2016). A new, dynamic era for somatic cell nuclear transfer?. Trends Biotechnol..

[47] Yang X., Smith S.L., Tian X.C., Lewin H.A., Renard J.P., Wakayama T. (2007). Nuclear reprogramming of cloned embryos and its implications for therapeutic cloning. Nat. Genet..

[48] Kim J. (2008). Chemosensitization prevents tolerance of Aspergillusfumigatus to antimycotic drugs. Biochem. Biophys. Res. Commun..

[49] Gray R., Dobson R. (2009). Extinct ibex is resurrected by cloning. Telegraph.

[50] Ammari A.A., Amran R.A., Al Ghadi M.G., Alhimaidi A.R. (2022). Morphometric Assessment of the Bovine Ovary for in vitro Matured Oocyte Quality to Determine Developmental Competence. Indian J. Anim. Res..

[51] Ammari A.A., ALghadi M.G., Alhimaidi A.R., Amran R.A. (2022). The role of passage numbers of donor cells in the development of Arabian Oryx–Cow interspecific somatic cell nuclear transfer embryos. Open Chem..

[52] Amarnath D., Choi I., Moawad A.R., Wakayama T., Campbell K.H. (2011). Nuclear-cytoplasmic incompatibility and inefficient development of pig-mouse cytoplasmic hybrid embryos. Reproduction.

[53] Melo L.M., Silva S.B., Magalhães L.C., Cortez J.V., Kumar S., Duarte J.M., Rola L.D., Chaves M.S., Freitas V.J. (2022). The use of somatic cell nuclear transfer to obtain interspecific cloned embryos from brown brocket deer karyoplast and bovine cytoplast: Embryo development and nuclear gene expression. Theriogenol. Wild.

[54] Srirattana K., Matsukawa K., Akagi S., Tasai M., Tagami T., Nirasawa K., Nagai T., Kanai Y., Parnpai R., Takeda K. (2011). Constant transmission of mitochondrial DNA in intergeneric cloned embryos reconstructed from swamp buffalo fibroblasts and bovine ooplasm. Anim. Sci. J..

[55] Chung Y., Bishop C.E., Treff N.R., Walker S.J., Sandler V.M., Becker S., Klimanskaya I., Wun W.S., Dunn R., Hall R.M. (2009). Reprogramming of human somatic cells using human and animal oocytes. Cloning Stem Cells.

[56] Wang K., Beyhan Z., Rodriguez R.M., Ross P.J., Iager A.E., Kaiser G.G., Chen Y., Cibelli J.B. (2009). Bovine ooplasm partially remodels primate somatic nuclei following somatic cell nuclear transfer. Cloning Stem Cells.

[57] Lagutina I., Lazzari G., Duchi R., Turini P., Tessaro I., Brunetti D., Colleoni S., Crotti G., Galli C. (2007). Comparative aspects of somatic cell nuclear transfer with conventional and zona-free method in cattle, horse, pig, and sheep. Theriogenology.

[58] Holt W.V., Pickard A.R., Prather R.S. (2004). Wildlife conservation and reproductive cloning. Reprodction.

[59] Li S., Li Y., Du W., Zhang L., Yu S., Dai Y., Zhao C., Li N. (2005). Aberrant gene expression in organs of bovine clones that die within two days after birth. Biol. Reprod..

[60] Ty L.V., Hanh N.V., Uoc N.T., Duc N.G., Thanh N.T., Bui L.C., Huu Q.X., Nguyen B.X. (2003). Preliminary results of cell cryobanking and embryo production of black bear (Ursus thibetanus) by interspecies somatic cell nuclear transfer. Theriogenology.

[61] Kitiyanant Y., Saikhun J., Chaisalee B., White K.L., Pavasuthipaisit K. (2001). Somatic cell cloning in buffalo (*Bubalus bubalis*): Effects of interspecies cytoplasmic recipients and activation procedures. Cloning Stem Cells.

[62] Malin K., Witkowska-Piłaszewicz O., Papis K. (2022). The many problems of somatic cell nuclear transfer in reproductive cloning of mammals. Theriogenology.

[63] Li J., Gao Y., Petkov S., Purup S., Hyttel P., Callesen H. (2014). Passagenumber of porcine embryonic germ cells affects epigeneticstatus and blastocyst rate following somatic cell nucleartransfer. Anim. Reprod. Sci..

